# Army Examinations and State License

**Published:** 1899-04

**Authors:** 


### "ARMY EXAMINATIONS AND STATE LICENSE."

UNDER the above title, the *Medical Record* makes editorial comment in its issue of March 18, 1899, that must have occasioned much surprise among its many readers and friends. It appears that a bill has been introduced into the legislature at Albany exempting medical officers who served during our war with Spain from examination for license to practise in this state.

The *Record* favors the bill as "a very commendable form of special legislation." A moment's reflection must convince any one interested that this bill must be aimed at the "relief" of some particular individual who seeks to obtain exemption from the examination now required by law under the plea of patriotism.

One might as well claim exemption from examination to enter the legal or clerical professions on the same grounds. A medical officer who served with the volunteers and after the war desired to enter the regular army medical staff corps, might with equal propriety claim exemption from the examination prescribed by the surgeon-general's office. To go further: a candidate for a college degree might with even greater propriety claim exemption by reason of medical service in the volunteer army.

It is a well-known fact that medical officers of volunteers in our war with Spain were given only at best a cursory examination, while contract surgeons were not examined at all until the war was practically over. It is, then, misleading for the *Record* to say in this relation that "an army medical board has a proverbially high standard of requirements." While the fact is true, it is not true that medical officers of volunteers were examined by the regular army board, or were expected to meet the regular army standard of requirements.

If "the young medical men recently graduated, who in consequence of enlisting into the military service during the late war" and who "were unable to appear for their regents' examination" had taken the examination prescribed for candidates for medical service in the regular army, no one could reasonably object to the proposition, even though it be absurd, that "by virtue of their army examination and subsequent service" they shall be entitled to a state license to practise.

Under the bill in question, however, a young man who graduated in a state requiring a much lower standard than New York, but who entered the volunteer medical military service, could claim license to practise without let or hindrance.

We have such a high regard for the *Record* and so generally agree with its position on questions relating to medical economy, that we are pained to dissent from its stand in this case. We fear that the editor-in-chief has either been misled by parties in interest, or that this editorial escaped his eye, for Dr. Shrady is a well-known advocate of higher medical education, as well as an opponent of special legislation on the subject. For these and other reasons we hope to see him recall the article in question and exert his influence to prevent legislation that is obviously subversive of the best interests of the profession, in which the distinguished editor of the *Record* is such a conspicuous leader.

---

SINCE the publication of the last number of the JOURNAL, the Pan-American exposition has become an assured fact. The men at the head of the enterprise, the board of directors, the different committees, are guarantees that the exposition will be a great success. The many attractions, the cheap railway rates, will bring people to Buffalo from the Atlantic as well as from the Pacific who will take advantage of cheap fares. It seems then that 1901 would be a good year for Buffalo to secure some if not many of the national medical and scientific societies. The American Medical Association could be induced perhaps to meet here and with the same push and energy among the physicians who intend to go to the Columbus meeting, as was evidenced by the Pan-American workers in Albany and Washington, should make a favorable impression and at its meeting next year carry it out to success.

THE January number of the *American Journal of Surgery and Gynecology*, published at St. Louis, is a woman's number. The idea of a woman's edition of a medical journal originated with the BUFFALO MEDICAL JOURNAL three years ago, the June number, 1896, being edited by the medical women of Buffalo. Our contemporary is rather late in the matter of a woman's edition, but the result is creditable to all concerned. All articles are the contribution of women physicians, many of them of more than national reputation. The name of Mary A. Dixon-Jones appears as associate editor. It is the contention of some who are not therefore to be considered unfriendly to medical women that they are unfitted by physical and mental endowment for successful competition with men in certain lines of medical work. These doubtless should read the record of the work of women as here presented. The success or failure of women physicians seems to rest largely with the community in which they work. The provincialism and conservatism of many eastern cities makes it impossible for a woman to succeed in any marked degree in such a specialty as surgery. Those fortunate women who practise among a people of more liberal and progressive spirit do not find sex a bar to the successful practice of abdominal surgery—that branch which perhaps more than any other requires boldness and initiative. The work of medical women will probably always be limited to a certain degree by sexual considerations. The patients who most naturally come to a woman are those of her own sex, but aside from this there is no question of her ability to do with like judgment and skill whatever an equally qualified practitioner of the opposite sex would attempt.

